# Rivaroxaban compared to no treatment in ER-negative stage I–III early breast cancer patients (the TIP Trial): study protocol for a phase II preoperative window-of-opportunity study design randomised controlled trial

**DOI:** 10.1186/s13063-020-04675-7

**Published:** 2020-08-27

**Authors:** John Castle, Emma Blower, Nigel J. Bundred, James R. Harvey, Jecko Thachil, Andrea Marshall, Karina Cox, Silvia Cicconi, Chris Holcombe, Carlos Palmieri, Cliona C. Kirwan

**Affiliations:** 1 Manchester Cancer Research Centre, The University of Manchester, Wilmslow Road, Manchester, M20 4GJ UK; 2 The Nightingale Centre, Wythenshawe Hospital, Manchester, M23 9LT UK; 3 Department of Haematology, Manchester Royal Infirmary, Manchester, M13 9WL UK; 4 Warwick Clinical Trials Unit, University of Warwick, Coventry, CV4 7AL UK; 5 Department of Breast Surgery, Maidstone Hospital, Maidstone, ME16 9QQ UK; 6 Cancer Research UK Liverpool Cancer Trials Unit, Liverpool, L69 3GL UK; 7 Breast Unit, Royal Liverpool and Broadgreen University Hospitals NHS Trust, Liverpool, L3 9TA UK; 8 Department of Molecular and Clinical Cancer Medicine, Liverpool, L69 3GA UK

**Keywords:** Tissue Factor, Thrombin, FXa, DOAC, NOAC, Rivaroxaban, Breast cancer, Clinical trial, Ki67

## Abstract

**Background:**

Breast cancer patients are at a four-fold increased risk of developing a venous thromboembolism (VTE), a major cause of death in this group. Conversely, coagulation factors promote tumour growth and metastasis. This has been evidenced in preclinical models, with an inhibitory effect of anticoagulants on cancer growth through proliferative, angiogenic, apoptotic, cancer stem cell and metastatic processes. The extrinsic clotting pathway is also more upregulated in patients in the relatively poorer prognosis oestrogen receptor (ER)-negative breast cancer subgroup, with increased tumour stromal expression of the coagulation factors Tissue Factor and thrombin.

Rivaroxaban (Xarelto®, Bayer AG, Leverkusen, Germany) is a direct oral anticoagulant (DOAC). It is a Factor Xa inhibitor that is routinely prescribed for the prevention of stroke in non-valvular atrial fibrillation and for both VTE prophylaxis and treatment. This trial will assess the anti-proliferative and other anti-cancer progression mechanisms of Rivaroxaban in ER-negative early breast cancer patients.

**Methods:**

This UK-based preoperative window-of-opportunity phase II randomised control trial will randomise 88 treatment-naïve early breast cancer patients to receive 20 mg OD Rivaroxaban treatment for 11 to 17 days or no treatment. Treatment will be stopped 24 h (range 18–36 h) prior to surgery or repeat core biopsy. All patients will be followed up for 2 weeks following surgery or repeat core biopsy.

The primary endpoint is change in tumour Ki67. Secondary outcome measures include tumour markers of apoptosis and angiogenesis, extrinsic clotting pathway activation and systemic markers of metastasis, tumour load and coagulation.

**Discussion:**

Laboratory evidence supports an anti-cancer role for anticoagulants; however, this has failed to translate into survival benefit when trialled in patients with metastatic disease or poor prognosis cancers, such as lung cancer. Subgroup analysis supported a potential survival benefit in better prognosis advanced disease patients. This is the first study to investigate the anti-cancer effects of anticoagulants in early breast cancer.

**Trial registration:**

UK National Research Ethics Service (NRES) approval 15/NW/0406, MHRA Clinical Trials Authorisation 48380/0003/001-0001. The sponsor is Manchester University NHS Foundation Trust, and the trial is co-ordinated by Cancer Research UK Liverpool Cancer Trials Unit (LCTU). EudraCT 2014-004909-33, registered 27 July 2015. ISRCTN14785273.

## Background

Breast cancer in women is associated with a four-fold increased risk of venous thromboembolism (VTE) compared to age-matched women without cancer [1, 2]. Chemotherapy-induced VTE occurs in up to 7% of early breast cancer and 17% of metastatic breast cancer patients [3]. Breast cancer patients who develop VTE have a worse survival, which cannot solely be accounted for by VTE-induced mortality [4, 5]. It is thought to in part be due to a promotion of cancer growth and metastasis by coagulation factors.

**Fig. 1:**
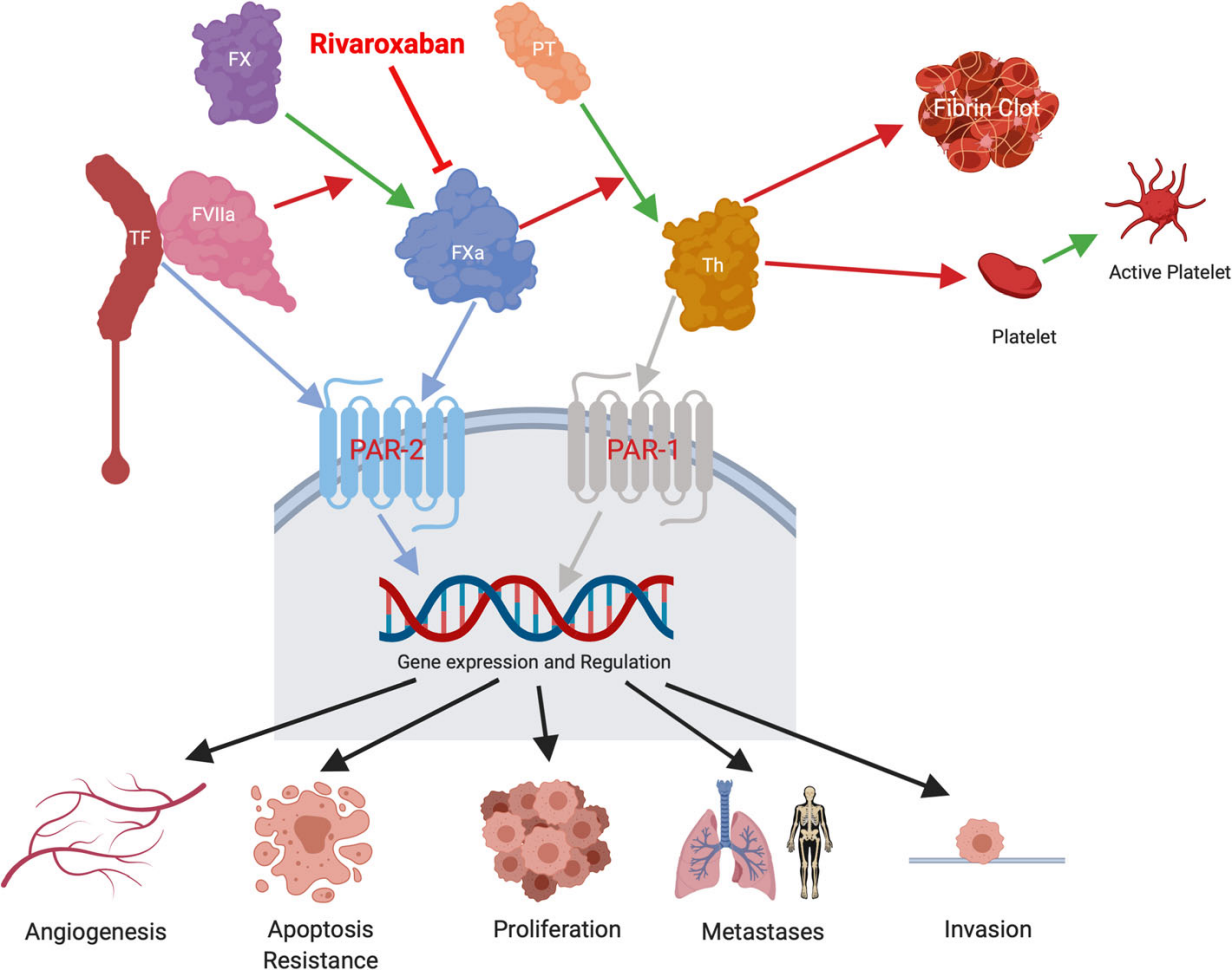
The extrinsic coagulation pathway. Tissue Factor (TF), the main initiator of the coagulation cascade, complexes with Factor VIIa to activate Factor X, and in turn converts prothrombin (PT) to thrombin (Th) and ultimately fibrinogen to fibrin, with subsequent clot formation. FXa is inhibited by the direct oral anticoagulant Rivaroxaban. Thrombin also binds to protease-activated receptor 1 (PAR-1), and the TF/FVIIa/Xa complex to PAR-2. In cancer cells, this is proposed to result in pro-angiogenic, pro-proliferative and pro-invasive gene expression resulting in increased metastases. Figure created with BioRender.com [6]. Abbreviations: TF, Tissue Factor; FVIIa, activated Factor VII; FX, Factor X; FXa, activated Factor X; PT, prothrombin; Th, thrombin; PAR, protease-activated receptor

Tissue Factor (TF), a transmembrane glycoprotein, is the initiator of the extrinsic clotting cascade. TF binds to plasma factor VII/VIIa (FVII/VIIa) to initiate coagulation via activation of Factor X (FX). This leads to the generation of thrombin, fibrin deposition and clot formation, as well as platelet activation (Fig. 1). In addition to its haemostatic role, TF activates cell signalling through TF:FVIIa and TF:FVIIa:FXa cleaving of the G-coupled protease-activated receptor (PARs) 1 and 2 complexes.

TF directly promotes tumour growth, migration and angiogenesis via activation of the PAR-2 receptor [7–11]. Thrombin can also modify tumour cell behaviour, directly through the activation of PAR receptors, indirectly by acting on stromal cells, or by the generation of fibrin matrices [12–14]. Thrombin promotes tumour growth in vitro [15] and in vivo [16–18], enhances tumour adhesion to platelets [16, 17, 19] and endothelial cells [20] in vitro and increases experimental (tail-vein injection) metastasis [16, 17, 19, 21]. Thrombin also increases tumour cell motility [22]. TF [23] and thrombin [24] promote neo-angiogenesis via upregulation of VEGF (a potent angiogenic factor) in cancer cells through the PI3K/Akt pathway [25]. TF also downregulates anti-angiogenic factors such as thrombospondin-1 and thrombospondin-2 [26], while thrombin upregulates matrix metalloproteinase-2 [27]. TF and thrombin also activate angiogenesis by clotting-dependent mechanisms through production (via thrombin) of polymerised fibrin which provides a scaffold around tumours to support and stimulate endothelial cell proliferation [28]. In clinical studies, TF expression correlates with VEGF expression and markers of angiogenesis [29], while prothrombin fragments (produced when prothrombin is converted to active thrombin) closely associate with areas of neo-angiogenesis at the tumour-stroma interface [30, 31].

Cancer cells express high levels of TF, thrombin and PARs [32, 33]. Breast cancer expression of TF has been reported to be an independent predictor of overall survival [34]. Plasma D-dimer (fibrin degradation by-product) is increased in breast cancer and correlates with tumour stage, poor prognosis molecular phenotypes oestrogen receptor (ER) negative, human epidermal growth factor receptor 2 (HER-2) positive [35, 36], circulating tumour cells [37, 38] and reduced survival [39]. D-dimer also has potential as a pharmacodynamic biomarker for chemotherapy response [40].

In a prospective cohort study of 248 early breast cancer patients, we found that tumour fibroblast expression of TF was increased in patients with primary breast cancer as compared to both ductal carcinoma in situ (DCIS) and normal breast. We also found that fibroblast TF, thrombin, PAR-1 and PAR-2 expression were increased in patients with ER-negative, HER-2-positive and high Ki67 tumours [41].

Inhibition of this coagulation cascade could therefore be a therapeutic option in breast cancer patients. A Cochrane review in 2011 reported that parenteral anticoagulation (with either unfractionated heparin or low molecular weight heparin) for patients with cancer, who have no therapeutic or prophylactic indication for anticoagulation, resulted in a statistically and clinically significant reduction in mortality at 24 months. This demonstrates a potential anti-cancer effect of anticoagulants in patients with better prognosis metastatic disease [42].

Direct oral anticoagulants (DOACs) inhibit thrombin (e.g. dabigatran etexilate (Pradaxa®)) or its activating enzyme Factor Xa (e.g. Rivaroxaban). DOACs have come into regular clinical use in the last decade for the prevention of VTE in orthopaedic surgery and of stroke in non-valvular atrial fibrillation [43, 44]. DOACs are also used for the treatment of primary or recurrent VTE. DOACs target a specific single enzyme within the extrinsic clotting pathway unlike the more widespread effects of heparins and vitamin K antagonists (Warfarin). A meta-analysis of randomised controlled trials of VTE-treatment with DOACs in cancer patients has indicated they are as safe and effective as conventional treatments [45].

Rivaroxaban was the first orally active direct Factor Xa inhibitor and, through inhibition of the Tissue Factor-Factor VIIa-Factor Xa complex, inhibits the conversion of prothrombin to thrombin [46]. Rivaroxaban is well absorbed from the gut and maximum inhibition of Factor Xa occurs 1 to 4 h after administration [47, 48]. Rivaroxaban has shown comparable safety to heparin for thromboprophylaxis after total hip replacements, with the convenience of oral dosing and reduced coagulation monitoring [49]. Rivaroxaban has the potential to downregulate the TF-FVIIa-FXa complex activation of PAR-2 and the subsequent tumour cell migration and production of pro-angiogenic and immune-modulating cytokines, chemokines and growth factors [10]. Rivaroxaban was chosen as the DOAC for this window-of-opportunity study as it inhibits the coagulation cascade at its origin and has a good safety profile. We have also found that Rivaroxaban inhibits cancer stem cell (CSC) activity in the in vitro functional CSC assay of mammosphere formation [50].

In summary, in vitro and in vivo evidence supports a potential inhibitory effect of anticoagulants on cancer growth through proliferative, cancer stem cell, angiogenic and metastatic processes. ER-negative early breast cancer patients have a relatively poorer prognosis and higher stage at diagnosis with an associated reduced disease-free survival. ER-negative early breast cancer patients also have fewer options for adjuvant therapy. Our previous data supports the hypothesis that the extrinsic clotting pathway is more upregulated in the ER-negative breast cancer subgroup as compared to ER-positive breast cancer, and thereby provides a potential therapeutic strategy.

To test a novel therapy in the adjuvant breast cancer setting would require thousands of patients, long follow-up and great expense. The alternative is the more novel ‘window-of-opportunity’ clinical trial design where drugs are tested over a short period, in the preoperative setting. Biomarkers are used as early markers for response and long-term outcome. Ki67 (tumour proliferation marker) is recognised as such a marker in trials of endocrine therapy. Short-term neoadjuvant studies have demonstrated that only 2 weeks of preoperative endocrine therapy induces changes in Ki67 which predicts for treatment benefit and long-term survival outcome [51]. By using biomarkers, we may be able to demonstrate the potential effectiveness of an anticoagulant in the clinical setting to support future larger adjuvant trials.

The TIP Trial will assess the anti-proliferative and other anti-cancer progression mechanisms of Rivaroxaban in ER-negative early breast cancer patients.

## Methods

This study protocol has been reported in accordance with the Standard Protocol Items: Recommendations for Clinical Interventional Trials (SPIRIT) guidelines (Additional file 1).

### Aim

The primary aim of the Thrombin Inhibition Preoperatively in Early Breast Cancer (TIP) trial is to determine whether preoperative administration of the oral Factor Xa inhibitor Rivaroxaban results in inhibition of tumour proliferation markers, as determined by a reduction in tumour Ki67 from baseline (pre-treatment) to post treatment (‘end-of-trial’, at time of surgical excision or repeat core biopsy prior to neoadjuvant chemotherapy), in ER-negative early breast cancer patients.

### Study design

The TIP trial is a phase II preoperative window-of-opportunity randomised controlled trial of Rivaroxaban compared to no treatment in ER-negative stage I–III early breast cancer patients.

This UK-based multi-centre study will randomise 88 eligible patients (1:1) to either Rivaroxaban 20 mg once daily (OD) or no treatment (control). Randomisation will be blinded to pathologists and data analysts, but not to patients or clinicians.

### Sample size calculation

The trial is designed with a two-sided alpha level of 0.05 and an estimated 80% power to detect a 0.6 standard deviation (SD) reduction in percentage tissue expression of Ki67 in early breast cancer patients treated with Rivaroxaban. Based on a two-sample *t* test with this 80% power, 88 eligible patients are required. We will allow for a 10% drop out rate, and therefore, 8 extra patients (giving a total of 96 patients) may need to be recruited. Any patient that withdraws from the trial or does not have surgery or a repeat core biopsy following treatment will be removed from the study and replaced.

### Participants

Females aged 18 years or over with newly diagnosed early-stage breast cancer (stages I–III) that have a tumour of size ≥ 10 mm and are oestrogen receptor negative (ER−) as defined by an ER QuickScore/Allred Score ≤ 5 will be eligible to participate in the TIP trial. Exclusion criteria include factors related to slow drug excretion (e.g. poor renal function), drug interactions (e.g. azole-antimycotics) and risk factors for bleeding (e.g. recent major surgery).

### Screening for eligibility

Assessments for patient eligibility to participate in the trial are completed prior to patient randomisation. These include blood biochemistry, liver function tests, baseline tumour assessment as per National Health Service Breast Screening Programme guidelines [52] and histopathological core biopsy including ER/PR receptor status. An electronic screening log is used to record each patient screened for the trial (even if not approached) and whether or not they were recruited.

### Randomisation

Patients will be randomly assigned (1:1) to either the control or intervention arm. Randomisation will be carried out centrally at the Liverpool Cancer Trials Centre (LCTC) based on pre-specified randomisation lists. The sequence of allocations is produced using the Stata package *ralloc* employing permutated block randomisation with block length of 4, 6 and 8. There are no stratification factors to be considered.

### Intervention group

Patients randomised to the intervention arm will receive Rivaroxaban 20 mg once daily for 11–17 days after diagnosis, prior to further treatment in the form of surgery or neo-adjuvant chemotherapy (NAC). Patients with planned NAC consent to undergo a repeat core biopsy once the trial treatment period has been completed.

**Fig. 2:**
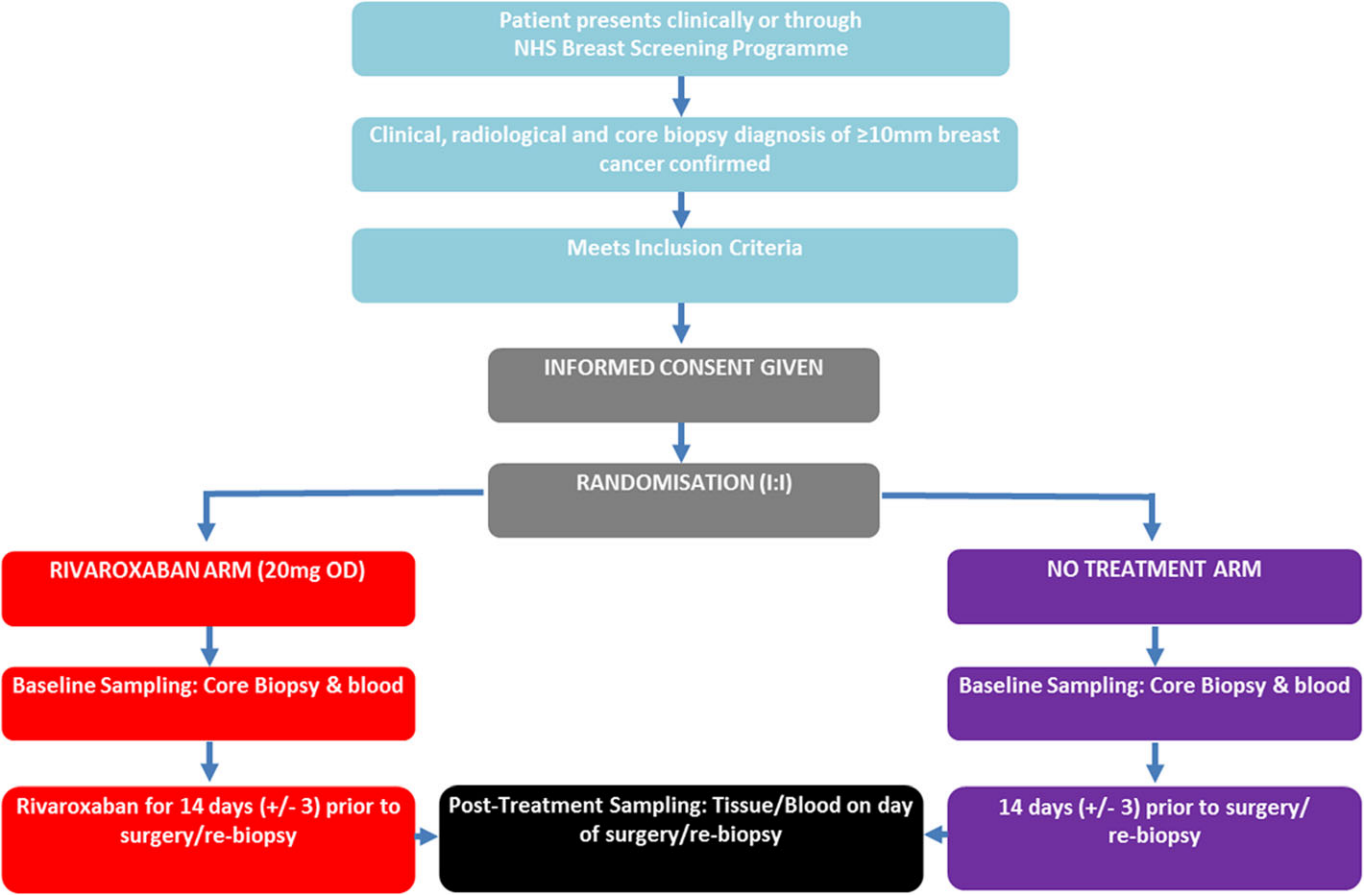
Thrombin Inhibition Preoperatively (TIP) in Early Breast Cancer Trial flow diagram. In this multi-centre study patients are randomised 1:1 20 mg Rivaroxaban once daily (OD): No Treatment. Randomisation is blinded to pathologists, research laboratory staff and data analysts, but not to patients and clinicians

**Fig. 3:**
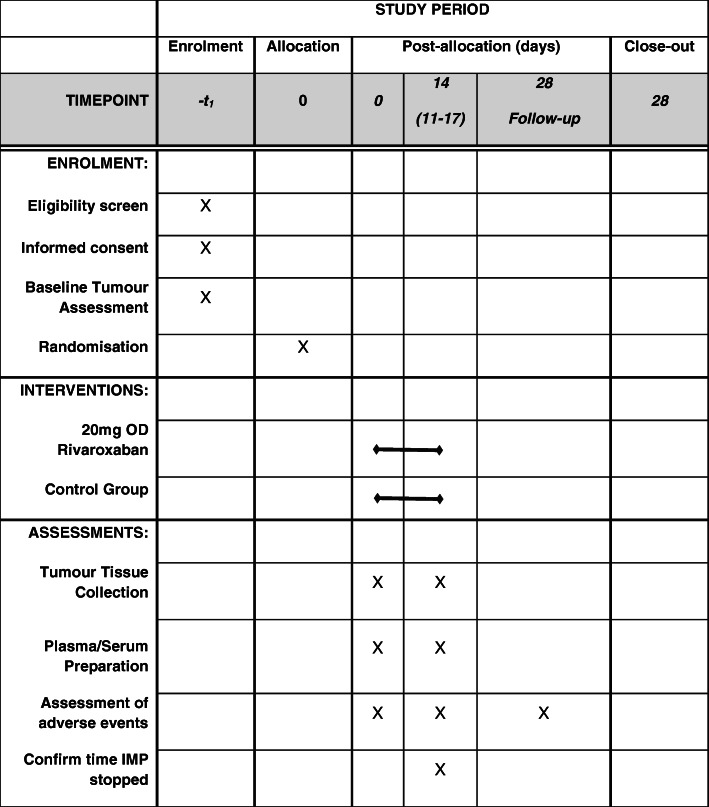
TIP Trial Standard Protocol Items: Recommendations for Interventional Trials (SPIRIT) Figure. Abbreviations: mg, milligram; OD, once daily

Rivaroxaban treatment is stopped 24 h (range 18–36 h) prior to surgery or repeat core biopsy before commencing NAC (Figs. 2 and 3). The dose of 20 mg OD was chosen as this is the licenced dose in the UK for the prevention of stroke and systemic embolism in adult patients with non-valvular atrial fibrillation with one or more risk factors [53]. No other concurrent neoadjuvant breast cancer or anticoagulant therapy is permitted.

All patients in the intervention arm will have an alert sticker placed in their notes to ensure that the medical team are aware that the patient has been anticoagulated and take all necessary precautions to minimise bleeding in the event of any residual anticoagulant effect.

Modifications to the rivaroxaban dose are not allowed; the intervention will simply be discontinued upon presentation with unacceptable toxicity.

### Control group

Patients randomised to the control group will not receive any anticoagulant therapy between diagnosis and either surgery or repeat biopsy prior to commencing NAC. They will receive current standard of care.

The use of a blinded placebo plus usual care comparator was considered in trial design but was decided against given the TIP trial has a biological endpoint (Ki67). Clinicians (surgeons or radiologists performing biopsies) are not to be blinded as a safety measure, but laboratory staff and data analysts will be blinded to avoid bias.

### Procedure

Following randomisation, all patients receive a copy of their signed consent form, a patient information sheet and a patient contact card. Residual tissue from the formalin-fixed paraffin-embedded pre-treatment diagnostic core biopsy tissue which is routinely stored will be requested for trial purposes and a 35-ml venous blood sample collected.

Upon termination of Rivaroxaban treatment or none, residual formalin-fixed and fresh tumour tissue will be obtained from trial patients undergoing surgery after routine clinical processing or from the repeat research biopsy prior to NAC.

All post-trial care, including provision for compensation, is through the NHS standard care pathway and NHS channels.

### Patient and public involvement

Patient and public representatives (PPI) were involved in the set-up of the trial, and a patient representative will take part in Trial Steering Committee meetings. PPI indicated to us that the trial risk was minimal and acceptable.

### Measures

Pathologists, research laboratory staff and data analysts will be blinded to patient randomisation in the TIP trial for assessment of both primary and secondary outcome measures.

### Primary outcome measure

#### Ki67

The primary outcome measure of the TIP trial is to determine the absolute and percentage change in the tumour tissue expression of the biomarker Ki67 before and after Rivaroxaban treatment, with the control arm acting as a reference group.

The completely specified primary outcome measure as defined by the five elements set out by Saldanha et al. [54]: *(1) Domain*: Breast cancer tumour cell proliferation. *(2) Specific measurement*: Immunohistochemical assessment of the proportion (%) of cells staining for the nuclear antigen Ki67. *(3) Metric*: Absolute and percentage change from baseline. *(4) Method of aggregation*: Mean. *(5) Time point*: Post-treatment (‘end-of-trial’, 14 days range 11–17, at time of surgical excision or repeat core biopsy prior to neoadjuvant chemotherapy).

### Secondary outcome measures

A range of potential breast cancer pharmacodynamic biomarkers will be studied as secondary outcome measures:

1. Primary mammosphere formation assays will be performed ex vivo on fresh tumour tissue at the end-of-trial time-point to determine cancer stem cell activity. This data will be presented as a comparison of the means of mammosphere forming efficiency (MFE) in the intervention and control arms. MFE is calculated by dividing the number of mammospheres formed at 7 days by the number of cells seeded [55].
2. Formalin-fixed paraffin-embedded (FFPE) tissue will be assessed for tumour markers of angiogenesis (CD31), apoptosis (Cleaved caspase 3, TUNEL) and the extrinsic clotting pathway (Tissue Factor, thrombin, PAR-1; in both epithelial cells/fibroblasts) by immunohistochemistry. Baseline core biopsy samples and end-of-trial biopsy samples will be assessed, and mean values analysed.
3. Plasma biomarkers of coagulation (Tissue Factor, thrombin and d-dimer) will be quantified by enzyme-linked immunosorbent assay (ELISA) with the change from baseline to the end-of-trial time-point assessed. Mean biomarker will be compared between the control and intervention arms.
4. FFPE tissue and plasma will be stored for future translational research, for example ctDNA, with patient consent.

Trial tumour tissue, plasma and serum samples, both for short-term storage and for future translational research, will be stored in temperature-controlled facilities at the Oglesby Cancer Research Building in Manchester, UK. This patient material will all be stored according to Good Clinical Laboratory Practise (GCLP) guidelines [56].

### Analysis

Both primary and secondary analyses will be carried out on an intention to treat (ITT) basis. Missing data or missing patients’ samples will be minimised by active monitoring by the Trial Coordinator, regularly reviewed by the Trial Management Group (TMG), including scheduled data cleaning, sample checking and communications with trial sites. If samples for the primary endpoint analysis are not collected, the patient will be replaced. Any patients who withdraw from the trial will be replaced. If the level of missing data exceeds these safeguards, it may be handled by multiple imputation. Separate multiple imputations by treatment arm would be performed, using the method of chained equations.

#### Ki67

Manual Section Score Ki67 will be compared between arms using a beta regression model including terms accounting for baseline Ki67, treatment arm, and covariates known to be associated with Ki67 such as tumour grade and HER-2 status. Although the sample size is based on a *t* test, a beta regression model for the analysis is superior, partly because the analysis would be on the original scale, partly because covariates including baseline would be easily accounted for and partly because treatment effects on variance could be explicitly modelled. Ki67 measurements will be performed in accordance with national guidance [57].

### Data management

The paper Case Report Form (CRF) distributed by the LCTU is the primary data collection instrument for the study and is completed by delegated site staff. The accuracy of these CRFs are the responsibility of the Principal Investigator at each site. CRF data is entered into an electronic database manually from the paper forms by LCTU staff. There is central monitoring by the LCTU to ensure that any data collected is compliant with the trial protocol. The database used for this trial includes validation features which will alert the user to certain inconsistent or missing data on data entry. Data stored at the LCTU will be checked for missing or unusual values (range checks) and checked for consistency over time. The LCTU will preserve the confidentiality of participants taking part in the study. Access to the final trial dataset is granted by the written authorisation of the Chief Investigator, CCK.

### Participant retention and complete follow-up

The Trial Coordinator will produce reports on patient withdrawals, losses to follow-up and the quantity of missing CRFs/data across sites for review by LCTU business meetings, the Trial Management Group (TMG), Trial Steering Committee (TSC) and Independent Safety and Data Monitoring Committee (ISDMC). Any problems identified will be discussed and remedial action taken as necessary. Patients who withdraw from the trial will be replaced, and any who deviate will be managed on a case-by-case basis.

If a patient wishes to withdraw from trial treatment, this will be acted upon immediately and will cause no detrimental effect to them in receiving usual standard of care. Following end of study biopsy, a single safety follow-up is performed at ~ 2/52. There is no long-term follow-up within the trial. All long-term follow-up will be as per usual NHS practice.

### Trial governance

#### Trial Coordinator (LCTU)

The LCTU will conduct central monitoring to ensure patient safety and that the trial procedures, trial intervention administration and laboratory and data collection processes meet both Sponsor and regulatory requirements. This includes ensuring that all regulatory and ethical approvals are in place before a site is opened, and generation of periodic Central Monitoring Reports for review by the Trial Management Group (TMG). The LCTU will carry out audits of trial sites and laboratories independent of the investigators and the Sponsor using set procedures after the first patient is recruited and after the tenth patient is recruited. The LCTU has a formalised Quality Management System in place for trial Quality Assurance.

#### Trial Management Group

The Trial Management Group (TMG) will comprise of the Chief Investigator, other lead investigators (clinical and non-clinical) and members of the LCTU. The TMG will be responsible for the day-to-day running and management of the trial and will meet approximately three times per year.

#### Trial Steering Committee

The Trial Steering Committee (TSC) includes a minimum of an independent Chairman; two additional independent expert members in the field of breast cancer (one being a statistician) and a lay/consumer representative, along with members of the Trial Management Group (TMG). Among other things, the TSC takes responsibility for monitoring and supervising the progress of the trial, considering recommendations from the Independent Safety and Data Monitoring Committee (IDSMC) and advising the TMG on all aspects of the trial as outlined in the TSC charter. Further details on the TIP Trial TSC charter can be obtained from the Trial Coordinator (https://www.lctu.org.uk).

The ultimate decision for the continuation of the trial lies with the TSC, and the TSC may stop the trial before completion upon recommendation of the IDSMC.

#### Independent Data and Safety Monitoring Committee

The TIP Trial Independent Safety and Data Monitoring Committee (IDSMC) is a multidisciplinary group independent from the Sponsor and competing interests. The IDSMC consists of an independent chairperson with breast cancer and/or significant trial experience, plus two further independent members: one statistician and at least one clinician. They are responsible for safeguarding the interests of trial participants, with interim monitoring of the safety and efficacy of the interventions during the trial, and for monitoring the overall progress and conduct of the clinical trial as outlined in the IDSMC charter. Further details on the TIP Trial IDSMC charter can be obtained from the Trial Coordinator.

The IDSMC will first convene prior to the recruitment of the first patient and will then define the required frequency of subsequent meetings (at least annually). The IDSMC will provide a recommendation to the Trial Steering Committee concerning the continuation of the study.

### Safety monitoring

Rivaroxaban is an oral anticoagulant, with an associated risk of bleeding, as with any anticoagulant. Unlike Warfarin, blood monitoring is not required. We will systemically collect data on all expected and unexpected adverse events at each study visit, with Principal Investigators required to assess and report them to the Trial Coordinator throughout the whole trial process, from trial entry through to follow-up. An adverse event term must be provided for each adverse event, using the terms listed in the Common Terminology Criteria for Adverse Events v4.03 [58]. Investigators must report serious adverse events (SAEs) within 24 h of the local site becoming aware of the event. Adverse events related to the study intervention will be reported in trial publications.

This study may be terminated at the request of the Chief Investigator, Independent Data and Safety Monitoring Committee or the Independent Ethics Committee if, during the course of the study, concerns about the safety emerge. Serious adverse events are relayed directly to the Chief Investigator via the Trial Coordinator, with all adverse events included in the Central Monitoring Reports reviewed at Trial Management Group meetings. Interim monitoring of safety is also carried out by the IDSMC. The report of a serious adverse reaction or multiple adverse reaction incidences (depending on severity) would constitute grounds for trial termination.

### Dissemination

We aim to present the data at scientific and medical conferences and to publish the results in a peer-reviewed journal. Authorship guidelines will be as per Recommendations for the Conduct, Reporting, Editing and Publication of Scholarly work in Medical Journals, International Committee of Medical Journal Editors (ICMJE) [59]. We have no intended use of professional writers.

## Discussion

Ki67 is recognised as a marker of tumour proliferation in trials of endocrine therapy. Short-term neoadjuvant studies have demonstrated that only 2 weeks of preoperative endocrine therapy induces changes in Ki67 which predicts for treatment benefit and long-term survival outcome [51]. By using biomarkers, we will be able to demonstrate the effectiveness of Rivaroxaban in the clinical setting within 5 years, thereby providing an improved clinical trials service to the patients recruited. However, the measurement of Ki67 has its challenges as there is substantial heterogeneity and variable levels of validity in methods of assessment [57]. It is agreed that we will use the immunohistochemistry (IHC) method for determining Ki67 percentages. The IHC staining and assessment of Ki67 will be performed on all trial samples in one batch upon trial completion in an attempt to minimise variability and increase accuracy.

The collection of fresh tumour tissue for mammosphere analysis will not always be feasible, particularly in patients with smaller tumours or who undergo a research biopsy. Mammosphere analysis is therefore a secondary and exploratory outcome of the TIP trial. However, if successful, it may allow assessment of the value of fresh tissue in ex vivo analysis as a biomarker of response.

This trial has been designed to minimise the chance of haematoma following surgery or biopsy in the presence of an anticoagulant. The terminal elimination half-life of Rivaroxaban is 5 to 9 h in healthy subjects aged 20 to 45 years and 11 to 13 h in elderly patients aged 60 to 76 years [60]. Clinical guidelines recommend stopping the drug in routine surgery patients at ≥ 24 h before surgery for minor risk procedures [61]; therefore, a safe time period, in patients with normal renal function, was taken as 24 h. However, clinicians (surgeons or radiologists performing biopsies) are still advised of the randomisation so bleeding precautions can be maximised.

The trial was initially designed as a window-of-opportunity pre-surgery study; however, with changing clinical practice, increasingly ER-negative patients are having neoadjuvant chemotherapy. Therefore, an amendment to the study was submitted to allow inclusion of neoadjuvant patients, with the post-trial biopsy being an additional research biopsy. It is routine to undergo radiological clip placement prior to commencement of chemotherapy, to allow identification of the tumour bed in the presence of complete radiological response to NAC. Therefore, to minimise impact on trial patients, the post-trial biopsy is performed at the time of clip placement. This was assessed as acceptable by our patient and public collaborators.

### Trial status

Current protocol: Version 6, dated 10 October 2017

Important protocol modifications are communicated to relevant parties by the trial coordinator by direct communication and via the LCTU Portal www.lctu.org.uk.

Recruitment start: June 2016

Expected recruitment end: September 2020

Open trial centres: Wythenshawe Hospital (Manchester), Royal Bolton Hospital, Royal Liverpool University Hospital, North Manchester General Hospital, St James’s University Hospital Leeds, Arrow Park Hospital/Clatterbridge Cancer Centre (Wirral), Churchill Hospital (Oxford); all United Kingdom.

Trial sponsor contact: Hayley Brooks, Oncology Clinical Trials Manager, Manchester University NHS Foundation Trust, Hayley.brooks@mft.nhs.uk

## Supplementary information

**Additional file 1.** SPIRIT 2013 Checklist: Recommended items to address in a clinical trial protocol and related documents.

## Data Availability

Access to the final trial dataset will be granted by the written authorisation of the Chief Investigator, CCK.

## Abbreviations

ABS: Association of Breast Surgery
CD31: Cluster of differentiation 31
DOAC: Direct oral anticoagulant (synonym: NOAC)
DOI: Digital Object Identifier
ECCO: European Cancer Organisation
ELISA: Enzyme-linked immunosorbent assay
ER: Oestrogen receptor
EudraCT: European Clinical Trials Database Reference
FFPE: Formalin-fixed paraffin-embedded
FVII: Factor VII (seven)
FVIIa: Activated Factor VII (seven)
FX: Factor X (ten)
FXa: Activated Factor X (ten)
GP: General Practitioner
HER2: Human epidermal growth factor receptor 2
IDSMC: Independent Data and Safety Monitoring Committee
ISRCTN: International Standard Randomised Controlled Trials Number
ITT: Intension to treat
LCTC: Liverpool Cancer Trials Centre
LCTU: Cancer Research UK Liverpool Cancer Trials Unit
MCRC: Manchester Cancer Research Centre
MFE: Mammosphere Forming Efficiency
MG: Milligram
MHRA: Medicines and Healthcare Products Regulatory Agency
NAC: Neoadjuvant chemotherapy
NIHR: National Institute for Health Research
NOAC: Novel oral anticoagulant (synonym: DOAC)
NRES: National Research Ethics Service
OD: Once daily
PAR: (1/2) protease activated receptor
PPI: Patient and public input
PT: Prothrombin
REC: Research Ethics Committee
TF: Tissue Factor
TIP: Thrombin Inhibition Preoperatively in Early Breast Cancer
TMG: Trial Management Group
TSC: Trial Steering Committee
TUNEL: Terminal deoxynucleotidyl transferase-mediated dUTP nick-end labelling
VEGF: Vascular endothelial growth factor
VTE: Venous thromboembolism
